# Case Report: Severe neutropenia induced by avelumab in a patient with Merkel cell carcinoma

**DOI:** 10.3389/fimmu.2025.1672282

**Published:** 2025-10-09

**Authors:** Eri Ohta, Kayo Yamamoto, Hitomi Fujimura, Natsuko Saito-Sasaki, Etsuko Okada, Yu Sawada

**Affiliations:** Department of Dermatology, University of Occupational and Environmental Health, Kitakyushu, Japan

**Keywords:** avelumab, case report, Merkel cell carcinoma, neutropenia, irAE

## Abstract

A 78-year-old woman with a history of follicular lymphoma treated with obinutuzumab and bendamustine developed Merkel cell carcinoma (MCC) on her left forearm. After surgical excision, in-transit and lymph node metastases appeared within three weeks. Avelumab and radiotherapy were initiated, but severe neutropenia occurred after two doses, requiring G-CSF support and treatment discontinuation. Despite early cessation, metastatic lesions regressed. This case suggests that prior B-cell–directed therapy may increase the risk of hematologic toxicity with immune checkpoint inhibitors and that even short-term avelumab may induce a durable response in MCC.

## Introduction

Merkel cell carcinoma (MCC) is an aggressive cutaneous neuroendocrine tumor that primarily affects elderly or immunosuppressed individuals (1, 2). Despite its high propensity for recurrence and metastasis, immunotherapy such as avelumab has shown promising response rates and remains one of the few approved treatments for unresectable MCC (3). However, immune-related adverse events (irAEs) require careful management (4). Among these, hematologic toxicities such as neutropenia are rare, and only a limited number of cases have been reported. Here, we present a unique case of MCC complicated by severe neutropenia following avelumab therapy in a patient with a history of follicular lymphoma.

## Case report

A 78-year-old Japanese woman was referred to our dermatology department for evaluation of a rapidly enlarging, violaceous nodule on her left forearm. She first noticed a reddish papule approximately four months before presentation. Her medical history was notable for follicular lymphoma, diagnosed two years prior. She had undergone six cycles of chemoimmunotherapy with obinutuzumab and bendamustine, known as GB therapy. The treatment was completed approximately 90 days before her current dermatologic symptoms, and she had remained in complete remission without maintenance therapy. No severe hematologic toxicity was observed during GB therapy. She also had a history of paroxysmal atrial fibrillation and type 2 diabetes mellitus, both well controlled.

**Figure 1:**
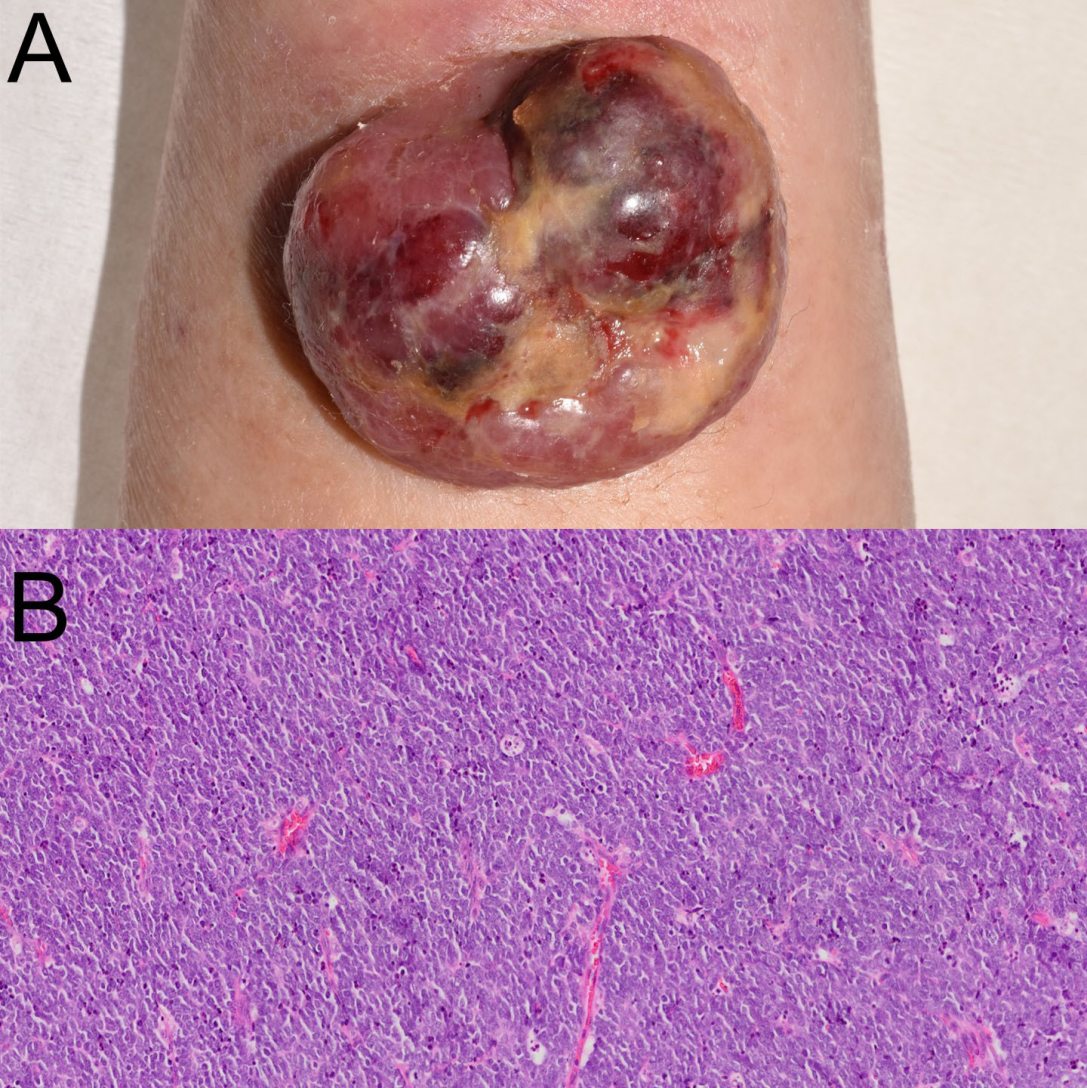
Clinical manifestation and histological examination. **(A)** Clinical image showing a violaceous, dome-shaped, ulcerated tumor on the left forearm. **(B)** Histopathologic examination revealed uniform small cells with high nuclear-to-cytoplasmic ratio.

On physical examination, a dome-shaped, reddish-purple tumor measuring 37 × 32 mm was observed on the radial side of the left forearm (**Figure 1A**). The surface showed ulceration with necrotic crusts. The lesion was firm, broad-based, and not fixed to deeper structures. Blood tests revealed mildly elevated lactate dehydrogenase (LDH) (382 U/L) and neuron-specific enolase (NSE; 19.8 ng/mL). Computed tomography (CT) from the neck to pelvis revealed no distant metastasis.

The lesion was excised with a 2 cm margin down to the level of the fascia. A full-thickness skin graft was taken from the lower right abdomen and transplanted to the skin defect. Histopathology revealed a nodular proliferation of small, round, hyperchromatic cells in the dermis (**Figure 1B**). Immunohistochemical staining showed diffuse positivity for CK20 in a perinuclear dot-like pattern, confirming the diagnosis of MCC and excluding recurrence of follicular lymphoma. Tumor cells exhibited perineural invasion, with horizontal progression in subcutaneous tissue, indicating in-transit metastasis. Final staging was pT2pN2M0, stage IIA.

Nineteen days postoperatively, a reddish nodule (17 × 10 mm) appeared to the graft margin confirmed recurrent MCC by skin biopsy. CT revealed subcutaneous nodules and axillary lymphadenopathy, indicating in-transit and regional metastases. Avelumab (10 mg/kg biweekly) and radiotherapy were initiated. Radiotherapy included 9 Gy/3 fractions to the forearm, and 30 Gy/15 fractions to the arm and axilla.

**Figure 2:**
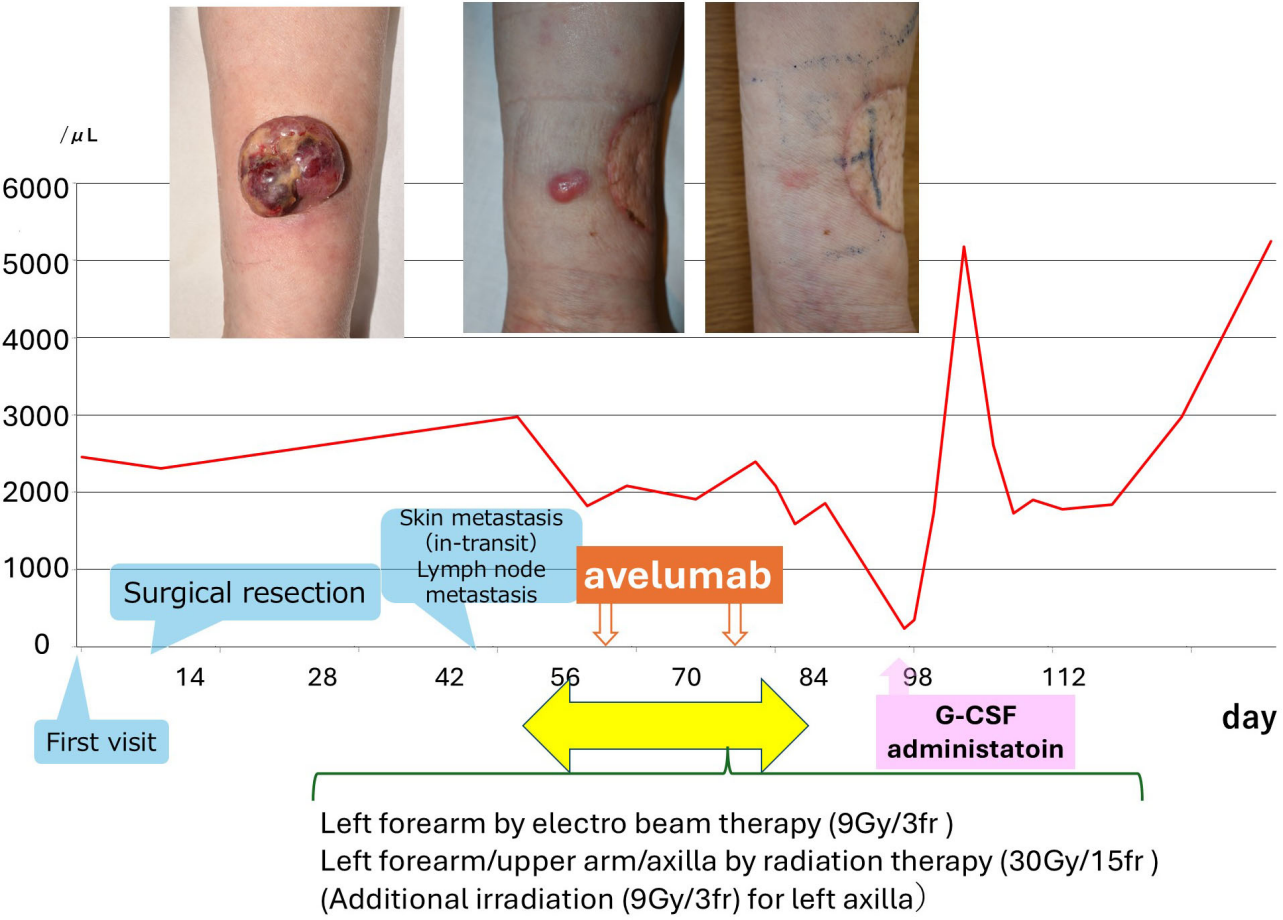
Clinical course and neutrophil count trend. The graph illustrates the timing of surgical resection, radiation therapy, avelumab administration, recurrence, and G-CSF treatment. Images at the top show the initial tumor, recurrent lesion, and post-treatment status. Notably, neutrophils declined significantly after the second avelumab dose and improved with G-CSF.

Avelumab was administered on days 0 and 14. On day 32 (18 days after the second dose), she developed fever and grade 4 neutropenia (233/μL) (**Figure 2**). G-CSF was administered for 3 days, leading to recovery with the discontinuation of avelumab. CT at two months showed regression of axillary lymphadenopathy.

## Discussion

Immune checkpoint inhibitors, especially avelumab, have become standard for advanced MCC. Although therapeutically effective for intractable malignant tumors, hematologic toxicity is rarely observed, and detailed management strategies have been infrequently reported. In our patient, severe neutropenia developed after only two doses of avelumab.

The mechanism of neutropenia induced by immune checkpoint inhibitors, particularly anti–PD-L1 antibodies such as avelumab, remains incompletely understood. Several hypotheses have been proposed: First, the PD-1/PD-L1 axis is not only involved in peripheral T-cell inhibition, but also plays a role in maintaining hematopoietic stem cell (HSC) quiescence within the bone marrow niche (5). Disruption of this signaling may impair granulopoiesis, particularly in patients with prior myelosuppressive therapy. Experimental studies have shown that PD-1 is expressed on HSCs, and its inhibition may shift the balance toward activation-induced exhaustion or apoptosis.

Second, immune checkpoint blockade may lead to aberrant activation of cytotoxic T lymphocytes against myeloid lineage cells, including mature neutrophils and progenitors (6). While anti-neutrophil antibodies were not detected in our case, T cell-mediated immune destruction remains a possible mechanism, as has been observed in other immune-related cytopenias.

Third, prior exposure to obinutuzumab and bendamustine likely created a vulnerable hematopoietic environment. She had previously received obinutuzumab and bendamustine for follicular lymphoma. Obinutuzumab induces strong antibody-dependent cellular cytotoxicity (ADCC) and direct cell death, while bendamustine is known to cause long-term bone marrow suppression and T-cell depletion. However, neutropenia was not observed during the period of caution following administration, and a direct causal relationship remains unclear. However, obinutuzumab exerts potent antibody-dependent cellular cytotoxicity and induces direct B-cell apoptosis, which may indirectly disrupt bone marrow immune homeostasis, including T-cell regulation (7). Bendamustine is known for delayed and prolonged T-cell depletion, which may modify the immune milieu and prime for dysregulated reconstitution under biologics (8). Notably, while no prior reports have described *de novo* MCC following obinutuzumab–bendamustine therapy, there has been a published case in which recurrent MCC developed during rituximab–bendamustine treatment for lymphoma (9). This suggests that profound B-cell depletion in combination with cytotoxic chemotherapy may not only predispose patients to hematologic vulnerability, but also impair immune surveillance and contribute to MCC progression. The convergence of these factors may have predisposed our patient to a severe, early-onset neutropenia after only two cycles of avelumab. This case highlights the need for careful monitoring of hematologic toxicity in patients receiving immune checkpoint inhibitors after B-cell–depleting therapies, and calls for further mechanistic studies to better predict and manage such adverse events.

Concurrent radiation was selected in addition to avelumab to improve local control and potentially enhance systemic immune responses, in line with NCCN guidelines. Although neutropenia is rarely reported with avelumab alone, the combination with radiotherapy may have had an additive myelosuppressive effect, and this possibility should be taken into account.

Notably, the tumor demonstrated rapid progression, with in-transit and lymph node metastases appearing only 19 days postoperatively. Such early recurrence may reflect an intrinsically aggressive tumor biology, though these were not assessed in our case. In addition, the patient received concurrent radiotherapy and avelumab, raising the possibility of a synergistic anti-tumor immune effect.

Despite early discontinuation of avelumab due to toxicity, the patient’s metastatic lymphadenopathy regressed over the following two months. This suggests that even a short course of immune checkpoint inhibition may be sufficient to induce durable anti-tumor immunity in selected patients.

## Conclusion

We report a case of MCC complicated by severe neutropenia following avelumab therapy, potentially exacerbated by prior obinutuzumab and bendamustine exposure. Clinicians should be aware of this rare but serious adverse event, particularly in immunocompromised patients or those with recent B-cell–directed therapy. Further research is needed to elucidate the mechanism and identify at-risk populations.

## Data Availability

The original contributions presented in the study are included in the article/supplementary material. Further inquiries can be directed to the corresponding author.
